# Body and Testicular Biometry and Epididymal Sperm Characteristics in Sambar Deer ( *Rusa unicolor* (Kerr, 1792))

**DOI:** 10.1111/rda.70122

**Published:** 2025-09-06

**Authors:** Isabella de Matos Brandão Carneiro, Rodrigo Freitas Bittencourt, Gleice Mendes Xavier, Eduardo Oliveira Costa, Amanda Íris dos Santos Correia, Miguel Ferreira Bomfim Baptista, Rodrigo Ribeiro Machado Mendes, Luiza Figueiredo Barbosa, Mateus Martins Rodrigues dos Santos, Luciano Cardoso Santos

**Affiliations:** ^1^ Department of Anatomy, Pathology and Veterinary Clinics School of Veterinary Medicine and Zootechnics, Federal University of Bahia (UFBA) Salvador Brazil; ^2^ Department of Veterinary Clinics School of Veterinary Medicine and Animal Science, São Paulo State University (Unesp) Botucatu São Paulo Brazil; ^3^ Department of Biological Sciences State University of Santa Cruz (UESC) Ilhéus Bahia Brazil

**Keywords:** conservation, morphometry, *Rusa unicolor*, semen

## Abstract

Characterising body and reproductive morphometry and their association with epididymal sperm quality can contribute to the conservation of sambar deer ( *Rusa unicolor* ). Five adult males maintained in captivity at the Getúlio Vargas Zoobotanical Park (Salvador, BA, Brazil) were captured, anaesthetised, and subjected to bilateral orchiectomy as part of a population‐control strategy. Body measurements included head circumference, thoracic diameter, total length, withers height, and body weight. The length, width, thickness, and weight of the testes and epididymides were measured, and the gonadosomatic index was estimated. Spermatozoa were recovered from the epididymal tail by slicing and flotation, and their morphology, membrane integrity, and kinematic parameters were assessed using a computerised computer‐assisted semen analysis (CASA) system. Mean kinematic parameters were: total motility (80.61% ± 18.33%), progressive motility (54.95% ± 16.55%), average path velocity—VAP (60.58 ± 12.38 μm/s), and percentage of normal spermatozoa (77.80% ± 6.14%). Withers height showed significant positive correlations (*p* < 0.05) with most reproductive parameters, including testicular weight (*r* = 0.936), testicular volume (*r* = 0.936), testicular area (*r* = 0.878), epididymal thickness (*r* = 0.882), total sperm recovered (*r* = 0.939), progressive motility (*r* = 0.888), and percentage of normal spermatozoa (*r* = 0.968). Additionally, testicular volume, thickness, epididymal length, epididymal width, and epididymal thickness showed significant positive correlations (*p* < 0.05) with most of the sperm parameters studied. These findings provide important preliminary data for future investigations on the reproductive potential of this species.

## 1 Introduction

The family Cervidae includes more than 60 species, many of which are threatened or extinct in the wild. Among these is the sambar deer ( *Rusa unicolor* ), which is native to Asia and classified as vulnerable (International Union for Conservation of Nature and Natural Resources 2022). Its complex behavioural traits pose challenges for obtaining accurate data on its physiological, nutritional, and reproductive status—key factors for practical conservation efforts for this species (Dahlan and Dawend 2013; Peres et al. 2021).

Reproductive morphology, particularly body, testicular, and epididymal biometrics, indicates overall health and reproductive potential in males, as these parameters correlate directly with spermatogenesis and sperm quality (Chacur et al. 2014; Lacerda et al. 2022). When the epididymis is the last source of viable genetic material, these assessments are critical for improving selection and reproductive management, which are important for advancing assisted reproduction techniques in *Rusa unicolor* and, consequently, maintaining specimens in their native habitats (Murad et al. 2023; Silva et al. 2018). Moreover, these assessments may provide valuable insights into the conservation of other threatened cervid species (Martins et al. 2015; MMA 2022).

Despite the conservation importance of sambar deer, few reports have described their reproductive management, both in situ and ex situ, or particularly their reproductive morphology. Therefore, we evaluated the body, testicular, and epididymal biometrics of captive males and correlated them with sperm parameters from the epididymal cauda using computer‐assisted semen analysis (CASA) and the gonadosomatic index in the present study. These findings have potential applications in breeder selection, contributing to the development of improved reproductive strategies for species conservation.

## 2 Material and Methods

### 2.1 Ethical Aspects

All procedures were conducted in accordance with the guidelines of the Animal Use Ethics Committee (CEUA) of the Federal University of Bahia (Protocol No. 24‐2020) and with authorization from the Chico Mendes Institute for Biodiversity Conservation (ICMBio) (SISBIO Licence No. 76003).

### 2.2 Study Site

The study was conducted in the municipality of Salvador, Bahia, Brazil (latitude 12°58′16″ S and longitude 38°30′39″ W, elevation 8 m). This region has an average temperature of 24°C–26°C and annual rainfall of 1200–1400 mm. The study period spanned from July 2020 to May 2024. Cervids were obtained from the Getúlio Vargas Zoobotanical Park Reserve.

### 2.3 Animals

Five healthy adult male *Rusa unicolor* aged 2 to 4 years were used. The animals were housed in enclosures containing clay, soil, trees, and grass. Their diet consisted primarily of roughage, vegetables, fruits, leafy greens, feed, mineral supplements and water ad libitum.

### 2.4 Clinical Examination and Sterilisation

All animals underwent a clinical assessment of their general and reproductive health before an elective bilateral orchiectomy, which was indicated for population control in the enclosure. Orchiectomy was performed under general anaesthesia using a protocol combining intramuscular ketamine (5 mg/kg; Syntec 10%, Tecnologia Farmacêutica Aplicada à Medicina Veterinária, Brazil), xylazine (0.2 mg/kg; Syntec 2%, Tecnologia Farmacêutica Aplicada à Medicina Veterinária, Brazil), and midazolam (0.2 mg/kg; União Química, Farmacêutica Nacional S/A, Brazil) (Caulkett 1997).

**FIGURE 1:**
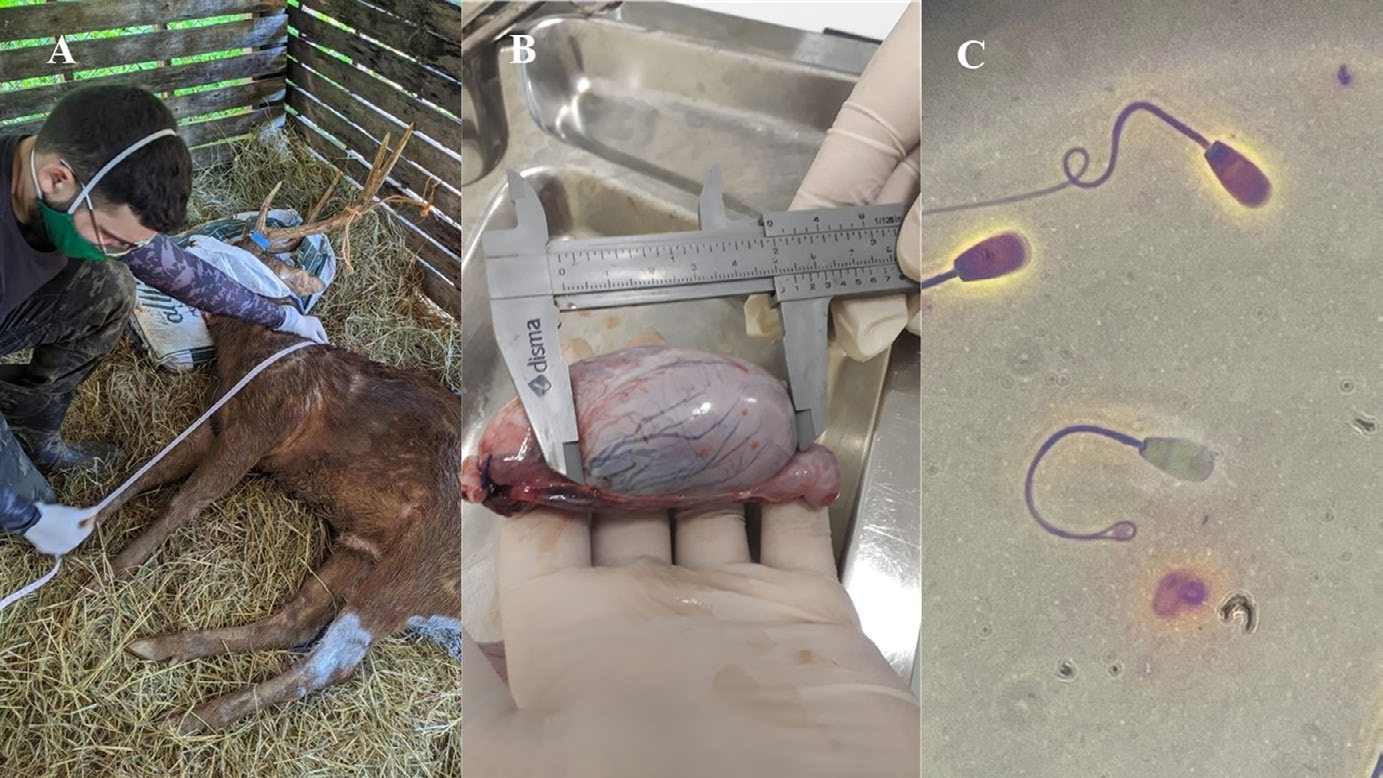
(A) Measurement of withers height of sambar deer. (B) Measurement of testicular length in sambar deer after orchiectomy. (C) Plasma membrane integrity by the supravital test (EOS) of sambar deer epididymal spermatozoa.

Body biometric parameters were measured using a tape measure (Figure 1). This included head circumference (HC), total length (TOL) (from the snout to the caudal end of the ischium), thoracic diameter (TD) (external circumference of the thoracic cavity, passing over the sternum and the spinous processes of the thoracic vertebrae), and withers height (WH) (from the highest point of the withers to the tip of the hoof) (Gurgel et al. 2022).

The body weight (BW) of the animals was estimated using the formula developed by Silva et al. (2006) for sheep, as follows: BW = −86.2391 + 1.5732TD (*r* ^2^ = 0.89).

The spermatic cord was ligated after sterilisation, and the testis–epididymis complexes were immersed in Ringer's lactate solution (NaCl, KCl, CaCl_2_, and NaC_4_H_5_O_3_) (Eurofarm, São Paulo, Brazil) at room temperature (22°C) for 10 min, and immediately transported to the laboratory (Martinez‐Pastor et al. 2006) in insulated containers.

### 2.5 Testicular Parameters and Seminal Evaluation

The testis–epididymis complexes were morphometrically evaluated before sperm collection and processing. Testicular width (TWI), thickness (TT), and length (TEL) were measured with callipers. Testicular area (TA) and volume (TV) were calculated using the equations proposed by Bailey et al. (1998), as follows: A = Π*r* ^2^ (*r* ^2^ = 1/2 length × 1/2 testicular width) and V = Π*r* ^3^ (r^3^ = 1/2 length × 1/2 width × 1/2 testicular thickness). Testicular weight (TWE) was measured using a 10‐g precision scale (Electronic, São Paulo, Brazil).

Based on the BW and the combined weight of both testes, the gonadosomatic index (GSI) was calculated as GW/BW × 100 (Amann 1970). Where GW is gonad weight (mean weight of the right and left testicles).

The epididymal width (EWI), thickness (ET), and length (EL) were measured using callipers, and the weight (EWE) was determined using a 10‐g precision scale (Electronic, São Paulo, Brazil).

Spermatozoa were obtained by epididymal sperm collection (Martinez‐Pastor et al. 2006). The testis–epididymis complexes (*n* = 5) were rinsed with Ringer's lactate. The epididymal cauda was isolated from the testes, and all adjacent connective tissues were carefully dissected. The epididymal tails were then placed in Petri dishes and maintained at room temperature (20°C–25°C) for dissection of superficial blood vessels and preparation of small cross‐sections. They were then soaked in a diluent medium of 3% Tris (hydroxymethyl) (Synth, Brazil), 20.00% (v/v) egg yolk, 1.05% (w/v) citric acid (Synth), 1.25% (w/v) glucose (VETEC, Brazil), 0.13% (v/v) gentamicin sulfate (Novafarma Produtos farmacêuticos, Brazil), and 0.50% (w/v) sodium lauryl sulfate (VETEC) in distilled water, pH 7.4, at room temperature for 30 min. The samples were transferred into 15 mL Falcon tubes and centrifuged (FANEM, São Paulo, Brazil) at 500 × *g* for 20 min.

After centrifugation, the supernatant was discarded, and the pellet was resuspended in the remaining diluent fraction, totaling approximately 0.5 mL. The total sperm recovery was assessed using a Neubauer chamber. Structural membrane integrity was evaluated using eosin staining (EOS), and functional integrity was evaluated using the hypoosmotic swelling test (HOST). The morphology was analysed in buffered saline under a phase‐contrast microscope (×100) and classified as minor or major defects (Colégio Brasileiro de Reprodução Animal (CBRA) 2013).

Sperm kinetics were evaluated using a CASA system (SCA, Microptics, Spain). An aliquot of the sample was placed on a pre‐warmed slide, covered with a coverslip (18 × 18 mm), and analysed under a phase‐contrast microscope (Eclipse 50i, Nikon, Japan, 100×), and images were captured using a video camera (Basler TM A312FC, Germany; setup in Table S2). Five random fields were selected per sample to assess total motility (TM), progressive motility (PM), curvilinear velocity (VCL), average path velocity (VAP), straight‐line velocity (VSL), straightness (STR), linearity (LIN), wobble (WOB), amplitude of lateral head displacement (ALH), and beat‐cross frequency (BCF).

### 2.6 Statistical Analysis

The data were analysed using GraphPad Prism 9 (version 9.5.0, build 730) and assessed for residual normality using the Shapiro–Wilk test (Razali and Wah 2011). Pearson's correlation was used for data with confirmed normal distribution (Table S1) (De Winter et al. 2016). Spearman's correlation was used to analyse the relationships between the body, testicular, epididymal, and seminal biometric parameters when at least one of the variables deviated from a normal distribution. Statistical significance was set at *p* < 0.05.

## 3 Results

Descriptive values for body, testicular, and tail epididymal biometrics in adult sambar deer are presented in Tables 1 and 2. The gonadosomatic index (GSI) showed an average value of 0.166%.

**TABLE 1:** Mean body biometric values of adult male Sambar deer (*n* = 5) undergoing orchiectomy.

Parameters	Mean	SD	SEM	Minimum	Q25	Median	Q75	Maximum
Head circumference (cm)	49	3.39	1.52	45	45.5	50	52	53
Thoracic diameter (cm)	100.8	6.54	2.92	94	94	102	107	107
Withers height (cm)	89.6	5.03	2.25	84	84.5	90	94.5	95
Total length (cm)	137.2	10.43	4.66	121	129	138	145	150
Body weight (kg)	72.4	10.04	4.49	62	62	74	82	82

Abbreviations: Q25, 25th quartile; Q75, 75th quartile; SEM, standard error of the mean.

**TABLE 2:** Mean testicular and tail of the epididymis biometry values and gonadosomatic index in adult sambar males (*n* = 5) undergoing orchiectomy.

Parameters	Mean	SD	SEM	Minimum	Q25	Median	Q75	Maximum
Testicular weight (g)	60.3	14.29	6.39	38.5	45.75	68.5	70.75	72.0
Testicular volume (cm^2^)	26.99	14.95	6.69	6.68	12.52	27.41	41.26	41.3
Testicular area (cm^2^)	14.9	5.09	2.28	7.20	9.72	17.46	18.81	19.17
Testicular width (cm)	3.55	0.89	0.40	2.25	2.63	4.00	4.25	4.30
Testicular thickness (cm)	2.46	0.76	0.34	1.35	1.80	2.35	3.17	3.23
Testicular length (cm)	5.22	0.78	0.35	4.00	4.60	5.20	5.85	6.10
Gonadosomatic index (%)	0.166	0.027	0.012	0.12	0.145	0.17	0.185	0.19
Epididymal weight (g)	3.17	1.30	0.58	1.37	1.82	3.73	4.25	4.50
Epididymal width (cm)	0.92	0.24	0.11	0.55	0.73	0.90	1.13	1.15
Epididymal thickness (cm)	0.92	0.20	0.09	0.65	0.75	0.85	1.13	1.15
Epididymal length (cm)	1.64	0.44	0.20	1.00	1.20	1.85	1.98	2.10

Abbreviations: Q25, 25th quartile 25; Q75, 75th quartile; SD, standard deviation; SEM, standard error of the mean.

Most testicular and epididymal caudal parameters showed positive and significant correlations (*p* < 0.05) with body measurements (Table 3; Figures S1 and S2). HC was correlated with TWE (*r* = 0.936, *p* = 0.019), TV (*r* = 0.889, *p* = 0.045), TA (*r* = 0.902, *p* = 0.035), TWI (*r* = 0.942, *p* = 0.016), EWE (*r* = 0.928, *p* = 0.03) and EL (*r* = 0.968, *p* = 0.0068). TD also correlated positively with TWE (*r* = 0.889, *p* = 0.043), TWI (*r* = 0.935, *p* = 0.019), EWE (*r* = 0.949, *p* = 0.013) and EL (*r* = 0.907, *p* = 0.033). BW significantly correlated with TWE (*r* = 0.885, *p* = 0.045), TWI (*r* = 0.932, *p* = 0.02), EWE (*r* = 0.947, *p* = 0.014), and EL (*r* = 0.905, *p* = 0.034).

**TABLE 3:** Correlations between body, testicular, and epididymal biometric measurements in adult male sambar deer (*n* = 5).

Parameters	Head circumference	Thoracic diameter	Withers height	Body weight
Testicular weight	0.936*	0.889*	0.854	0.885*
Testicular volume	0.883*	0.779	0.936*	0.773
Testicular area	0.902*	0.852	0.878*	0.847
Testicular width	0.942*	0.935*	0.816	0.932*
Epididymal weight	0.928*	0.949*	0.781	0.947*
Epididymal thickness	0.755	0.610	0.882*	0.602
Epididymal length	0.968**	0.907*	0.854	0.905*

* *p* < 0.05.

** *p* < 0.01.

The parameters for seminal analysis using conventional and computer‐assisted microscopy are presented in Table 4. Correlation analyses were performed between body, testicular, and tail biometrics of the epididymis and in natura seminal parameters to assess the potential effects of animal morphology on sperm quality (Table 5; Figures S3–S9).

**TABLE 4:** Sperm parameters of samples taken from the tail of the epididymis of adult male sambar deer (*n* = 5).

Parameters	Mean	SD	SEM	Minimum	Q25	Median	Q75	Maximum
Supravital test (EOS, %)	68.20	6.57	2.94	63.00	63.50	64.00	75.00	78.00
Hyposmotic test (HOST; %)	68.20	10.16	4.54	56.00	59.00	67.00	78.00	82.00
Total sperm recovered (×10^6^)	709.0	558.7	249.9	235.00	317.50	540.00	1385.00	1480.00
Total motility (%)	80.61	18.33	8.19	48.63	66.32	85.40	92.50	93.80
Progressive motility (%)	54.95	16.55	7.40	34.43	40.93	49.40	71.76	75.10
Average path velocity (μm/s)	60.58	12.38	5.53	50.10	51.08	52.66	74.04	75.78
Amplitude of lateral head displacement (μm/s)	3.57	0.35	0.17	3.28	3.285	3.52	3.928	3.99
Curvilinear velocity (μm/s)	99.61	16.92	7.56	81.67	84.6	96.65	116.1	124.0
Straight‐line velocity (μm/s)	34.93	8.89	3.97	24.54	26.8	35.47	42.79	47.78
Straightness (%)	57.25	7.86	3.51	47.72	50.32	55.06	65.28	66.67
VAP/VCL ratio (WOB)	61.67	6.47	2.89	56.21	57.23	59.00	66.21	72.49
Beat cross frequency (Hz)	8.30	1.58	0.70	6.30	7.06	8.03	9.685	10.65
Linearity (%)	37.79	8.94	3.99	28.51	30.27	35.55	46.43	51.21
Major defects (%)	8.40	4.34	1.93	4.00	5.00	6.00	13.00	14.00
Minor defects (%)	13.80	1.92	0.86	11.00	12.00	14.00	15.50	16.00
Normal (%)	77.80	6.14	2.74	70.00	71.50	80.00	83.00	85.00

Abbreviations: Q25, 25th quartile 25; Q75, 75th quartile; SD, standard deviation; SEM, standard error of the mean.

**TABLE 5:** Correlations between body, testicular, and tail of the epididymis biometrics and sperm parameters in male sambar deer (*n* = 5).

	Supravital test (EOS)	Hyposmotic test	Total conc.	Total motility	Progressive motility	Average path velocity	Major defects	Minor defects	Normal
Head circumference	0.302	−0.885*	0.736	1000*	0.835	0.589	−0.850	−0.651	0.804
Withers height	0.229	−0.937*	0.939*	0.600	0.888*	0.854	−0.953*	−0.940*	0.968**
Testicular volume	0.115	−0.822	0.943*	0.700	0.968**	0.838	−0.846	−0.823	0.855
Testicular weight	−0.008	−0.752	0.785	0.900	0.862	0.613	−0.837	−0.688	0.807
Testicular area	−0.069	−0.745	0.825	0.700	0.869	0.659	−0.861	−0.749	0.843
Testicular width	−0.031	−0.738	0.694	0.900	0.776	0.498	−0.837	−0.641	0.799
Testicular thickness	0.018	−0.708	0.932*	0.700	0.962**	0.839	−0.749	−0.770	0.770
Gonadosomatic index	−0.247	−0.488	0.77	0.615	0.836	0.633	−0.623	−0.596	0.626
Epididymal length	0.135	−0.797	0.788	0.974*	0.892*	0.633	−0.813	−0.655	0.780
Epididymal thickness	0.079	−0.728	0.968**	0.615	0.971**	0.905*	−0.742	−0.811	0.778
Epididymal width	0.045	−0.658	0.866	0.820	0.953*	0.765	−0.669	−0.649	0.676
Epididymal weight	−0.057	−0.717	0.623	0.900	0.700	0.413	−0.846	−0.607	0.788

* *p* < 0.05.

** *p* < 0.01.

The body measurement with the most significant effect was WH, which was significantly positively correlated with total sperm recovery (*r* = 0.939, *p* = 0.017), PM (*r* = 0.888, *p* = 0.044), and morphologically normal spermatozoa (*r* = 0.968, *p* = 0.006). Additionally, height was significantly and negatively correlated with major (*r* = −0.953, *p* = 0.011) and minor (*r* = −0.940, *p* = 0.017) defects.

Other notable correlations were observed between testicular and epididymal biometrics and sperm parameters. For example, total sperm recovery was strongly correlated with testicular volume (*r* = 0.943, *p* < 0.016) and testicular thickness (*r* = 0.932, *p* < 0.020). Progressive motility significantly correlated with testicular volume (*r* = 0.968, *p* < 0.006), testicular thickness (*r* = 0.962, *p* < 0.01), epididymal length (*r* = 0.892, *p* < 0.041), and epididymal thickness (*r* = 0.971, *p* < 0.005). The average path velocity (VAP) was also correlated positively with epididymal thickness (*r* = 0.905, *p* < 0.034).

Spearman's analysis (Table 6) revealed that although many correlations between the age of the animals and the studied parameters were strong, they did not reach statistical significance.

**TABLE 6:** Correlations between age and body biometrics, testicular, epididymal, and sperm parameters in male sambar deer (*n* = 5).

	Spearman *r*	*p*
Head circumference	0.866	0.200
Withers height	0.866	0.200
Thoracic diameter	0.912	0.200
Total length	0.000	> 0.999
Body weight	0.912	0.200
Testicular volume	0.866	0.200
Testicular weight	0.866	0.200
Testicular area	0.866	0.200
Testicular width	0.866	0.200
Testicular thickness	0.866	0.200
Testicular length	0.740	0.300
Gonadosomatic index	0.740	0.300
Epididymal length	0.888	0.100
Epididymal thickness	0.740	0.300
Epididymal width	0.740	0.300
Epididymal weight	0.866	0.200
Supravital test (EOS)	0.296	0.700
Hyposmotic test (HOST)	−0.866	0.200
Total sperm recovered	0.866	0.200
Total motility	0.866	0.200
Progressive motility	0.866	0.200
Average path velocity	0.288	0.800
Major defects	−0.888	0.100
Minor defects	−0.866	0.200
Normal morphology	0.866	0.200

## 4 Discussion

Adult sambar deer weigh between 117 and 320 kg in the wild, with WH and TOL ranging from 102–160–cm and 162–246 cm, respectively (Leslie Jr. 2011; Podchong et al. 2009; Watter et al. 2020). In captivity, younger animals (1–3 years) show lower values of 60.5–89 kg, 80–92 cm WH, and 124–143 cm TOL (Martins et al. 2015), which aligns with our findings for 2 to 4‐year‐olds.

Multiple factors, including age and growth chronology, may explain this discrepancy (Owens et al. 1993). However, considering that the animals in the present study are adults, it is more likely that such differences result from aspects of phenotypic plasticity and the lack of selective pressure in isolated populations (Crates et al. 2023; Mason et al. 2019). Captivity‐related factors such as management, nutrition, limited space, and chronic stress reflect an adaptive morphological response to the environment (Courtney Jones et al. 2018).

The relationship between body development and reproductive organ size is widely recognised in the literature and constitutes the basis for functional allometry. Larger individuals tend to exhibit proportional increases in specific body structures, including reproductive organs, particularly in the context of reproductive competition (Fernandes et al. 2005; Mysterud et al. 2001). This pattern is related to the growth curve, which guides the physical and functional maturation of an organism and is influenced by environmental and genetic factors (Owens et al. 1993).

We observed positive correlations between body measurements and testicular and epididymal dimensions in this study, reinforcing this allometric model. Body weight, withers height, thoracic diameter, and head circumference were significantly associated with gonadal morphometry, with the latter correlating with all evaluated parameters except epididymal thickness, which showed a high correlation coefficient (*r* = 0.755).

Although age is an important factor in the growth curve and, consequently, in the acquisition of reproductive potential (Chacur et al. 2014), the absence of a significant correlation between age and reproductive variables in this study suggested that the observed effects are closely related to the degree of individual body development. However, some variables showed strong correlations with age, although not statistically significant (*p* > 0.05), indicating a trend that deserves further investigation using larger sample sizes.

Withers height stood out because of its strong simultaneous correlations with testicular dimensions (volume, area, and thickness), epididymal measurements, and all evaluated sperm parameters (sperm recovery, motility, and morphology). These correlations suggested that body measurement may act as a marker of spermatogenic efficiency and hold particular promise as a selection criterion for individuals in reproductive management and assisted reproduction programmes.

Testicular measurements are strongly associated with reproductive activity and spermatogenic efficiency as they reflect the amount of functional parenchyma, especially the proportion of seminiferous tubules (Silva et al. 2018). A larger testicular mass often correlates with the number of germ and Sertoli cells, which are key elements in supporting and regulating sperm production (Montes‐Garrido et al. 2023; Souza et al. 2014).

These patterns supported the findings of the present study in which testicular size was positively associated with semen quality, reinforcing its role as a marker of male reproductive competence. In addition to spermatogenesis, more developed testes tend to exhibit greater androgen synthesis capacity, which directly influences the expression of sexual behaviours and the functionality of accessory reproductive structures (Kerketta et al. 2015; Masoud et al. 2021).

Correlations between epididymal measurements and sperm parameters further supported the functional role of this structure in maturation and acquisition of sperm motility (Monteiro et al. 2014; Raji et al. 2008). In the present study, in addition to a significant correlation between epididymal thickness and total sperm recovery, all epididymal measurements were positively associated (*p* < 0.05) with sperm kinematic parameters.

The mean gonadosomatic index (GSI) observed (0.16% ± 0.2%) was similar to that of *Cervus elaphus* (0.18%) and *Odocoileus virginianus* (0.19%), lower than that of *Mazama* spp. (0.4%), and higher than *Axis axis* (0.04%–0.09%) (Costa et al. 2011; Kenagy and Trombulak 1986; Willard and Randel 2002). These variations reflect differences in body size as well as genetic and environmental factors. Although GSI is widely used as an indicator of male reproductive investment (Pimenta et al. 2020; Raji et al. 2008), it did not show a significant correlation with semen quality in this study. Nevertheless, the high coefficients (*r* = 0.77, total sperm count, and *r* = 0.836, progressive motility) suggested an association trend that may have been limited by the sample size.

Despite the sample size limitation, the results help consolidate the patterns described in the literature and highlight the potential of body and gonadal morphometry as indicators of reproductive performance. In conservation and management contexts, such data support strategies for selecting breeders and may also provide insights into the nutritional and health status of wild populations.

In this study, the slicing and flotation technique proved effective in obtaining epididymal spermatozoa from sambar deer, yielding samples with quality compatible with the parameters described for ruminants and other cervids (Colégio Brasileiro de Reprodução Animal (CBRA) 2013).

The CASA system is essential for identifying detailed kinetic patterns, enabling a more accurate and standardised evaluation of sperm fertilisation potential (Tanga et al. 2021; World Health Organization 2021). However, considering the scarcity of sperm kinetic data in cervids and the lack of specific information on *Rusa unicolor* , this study reinforces its importance as an initial description of this species.

## 5 Conclusion

This study provides preliminary evidence of associations between testicular and epididymal tail measurements and sperm morphology and kinetics in *Rusa unicolor* . Body biometrics, particularly WH, appear to be related to reproductive parameters. Although limited by a small sample size, the observed patterns provide valuable descriptive data for the species and may inform future studies with larger samples. Additionally, the successful recovery of epididymal sperm using the slicing and flotation method demonstrates its potential utility for post‐mortem semen collection and biobanking in conservation programmes.

## Author Contributions

I.M.B.C., L.C.S. and R.F.B. designed the experiment, analysed the data and corrected the text; I.M.B.C., R.F.B., G.M.X., E.O.C., A.Í.S.C., M.M.R.S. collected the data; I.M.B.C., R.F.B., M.F.B.B., R.R.M.M. and L.F.B. wrote the original draft of the manuscript; R.F.B. and I.M.B.C. revised the final version of the manuscript.

## Conflicts of Interest

The authors declare no conflicts of interest.

## Supporting information


**Data S1:** rda70122‐sup‐0001‐DataS1.docx.

## Data Availability

The data supporting the findings of this study are available from the corresponding author upon reasonable request.
