# Supplementary material for: Body and Testicular Biometry and Epididymal Sperm Characteristics in Sambar Deer ( Rusa unicolor (Kerr, 1792))
Source: Reprod Domest Anim. 2025 Sep 6;60(9):e70122. doi: 10.1111/rda.70122 (PMC12413589; doi:10.1111/rda.70122)
Supplement: Supplementary file 1 — Data S1: rda70122‐sup‐0001‐DataS1.docx. [file RDA-60-e70122-s001.docx]

**Supplementary data**

**Table 1** - Results of normality tests for dependent and independent variables in male sambar deer (n=5).

| **Parameter** |  |  |  | **Shapiro-Wilk test** | |  |
| --- | --- | --- | --- | --- | --- | --- |
|  | **Probability normal (Gaussian)** | **Likelihood ratio (LR)** | **1/LR** | **W** | ***p*-value** | **Passed normality test (alpha=0.05)?** |
| Age | 55.35% | 1.240 | 0.806 | 0.6840 | 0.0065 | No |
| Head circumference | 50,51% | 1,021 | 0,9797 | 0,9252 | 0,5644 | Yes |
| Thoracic diameter | 50,52% | 1,021 | 0,9794 | 0,8122 | 0,1015 | Yes |
| Withers height | 50,20% | 1,008 | 0,9921 | 0,8887 | 0,3504 | Yes |
| Body weight | 50,90% | 1,037 | 0,9647 | 0,8143 | 0,1055 | Yes |
| Testicular weight | 60,82% | 1,552 | 0,6442 | 0,8430 | 0,1733 | Yes |
| Testicular volume | 65,51% | 1,900 | 0,5264 | 0,9100 | 0,4676 | Yes |
| Testicular area | 65,75% | 1,920 | 0,5208 | 0,8615 | 0,2338 | Yes |
| Testicular width | 59,39% | 1,462 | 0,6839 | 0,8584 | 0,2224 | Yes |
| Epididymal weight | 52,55% | 1,108 | 0,9029 | 0,9244 | 0,5617 | Yes |
| Epididymal thickness | 59,37% | 1,461 | 0,6843 | 0,9193 | 0,5256 | Yes |
| Epididymal length | 51,75% | 1,072 | 0,9324 | 0,9177 | 0,5155 | Yes |
| Epididymal width | 62,22% | 1,647 | 0,6072 | 0,8962 | 0,3894 | Yes |
| Gonadosomatic index | 61,29% | 1,583 | 0,6317 | 0,8202 | 0,1172 | Yes |
| Supravital test | 46,31% | 0,8627 | 1,159 | 0,8196 | 0,1159 | Yes |
| Hyposmotic test | 48,7% | 0,9492 | 1,053 | 0,9862 | 0,9648 | Yes |
| Total conc. (x10^6^) | 34,22% | 0,5202 | 1,922 | 0,87 | 0,2663 | Yes |
| Total motility | 68,11% | 2,136 | 0,4681 | 0,7509 | 0,0303 | No |
| Progressive motility | 49,75% | 0,9902 | 1,01 | 0,9401 | 0,6666 | Yes |
| Average path velocity | 44,77% | 0,8106 | 1,234 | 0,7855 | 0,0614 | Yes |
| Major defects | 38,78% | 0,6334 | 1,579 | 0,8713 | 0,2717 | Yes |
| Minor defects | 53,61% | 1,156 | 0,8653 | 0,9787 | 0,9276 | Yes |
| Normal morphology | 50,94% | 1,038 | 0,963 | 0,9391 | 0,6598 | Yes |

**Table 2** - The setup used for the assessment of sambar deer sperm kinetics using a system, an automatic sperm analyzer SCA®.

| **Parameters** | **Value** |
| --- | --- |
| Chamber depth | 12,5 |
| Minimum cell area (µm) | 5 |
| Maximum cell area (µm) | 80 |
| Static spermatozoon (µm/sec) | <10 |
| Slow–medium velocity spermatozoon (µm/sec) | 45 |
| Rapid spermatozoon (µm/sec) | >75 |
| Progressive spermatozoa (STR >) (µm/sec) | 80 |
| Connectivity | 12 |
| VAP (µm/sec) | 5 |

STR, straightness; VAP, average path velocity


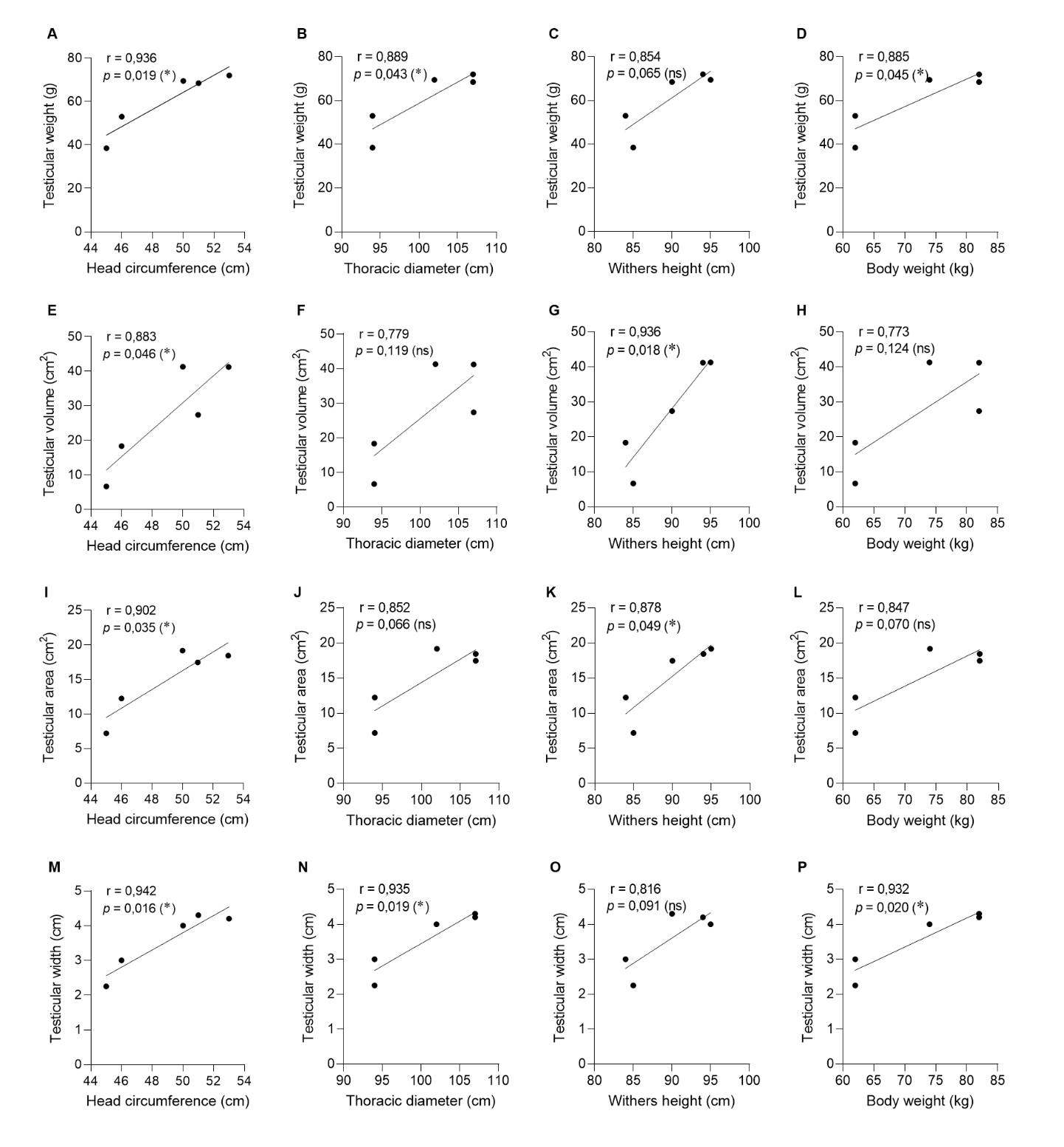


**Figure 1** - Correlations between body biometric measurements and testicular biometrics in adult male sambar deer. A-P) Correlations between testicular weight (A-D), volume (E-H), area (I-L) and width (M-P) with head circumference (A, E, I and M), thoracic diameter (B, F, J and N), withers height (C, G, K and O) and body weight (D, H, L and P) in adult male sambar deer. Legend: *p<0.05.


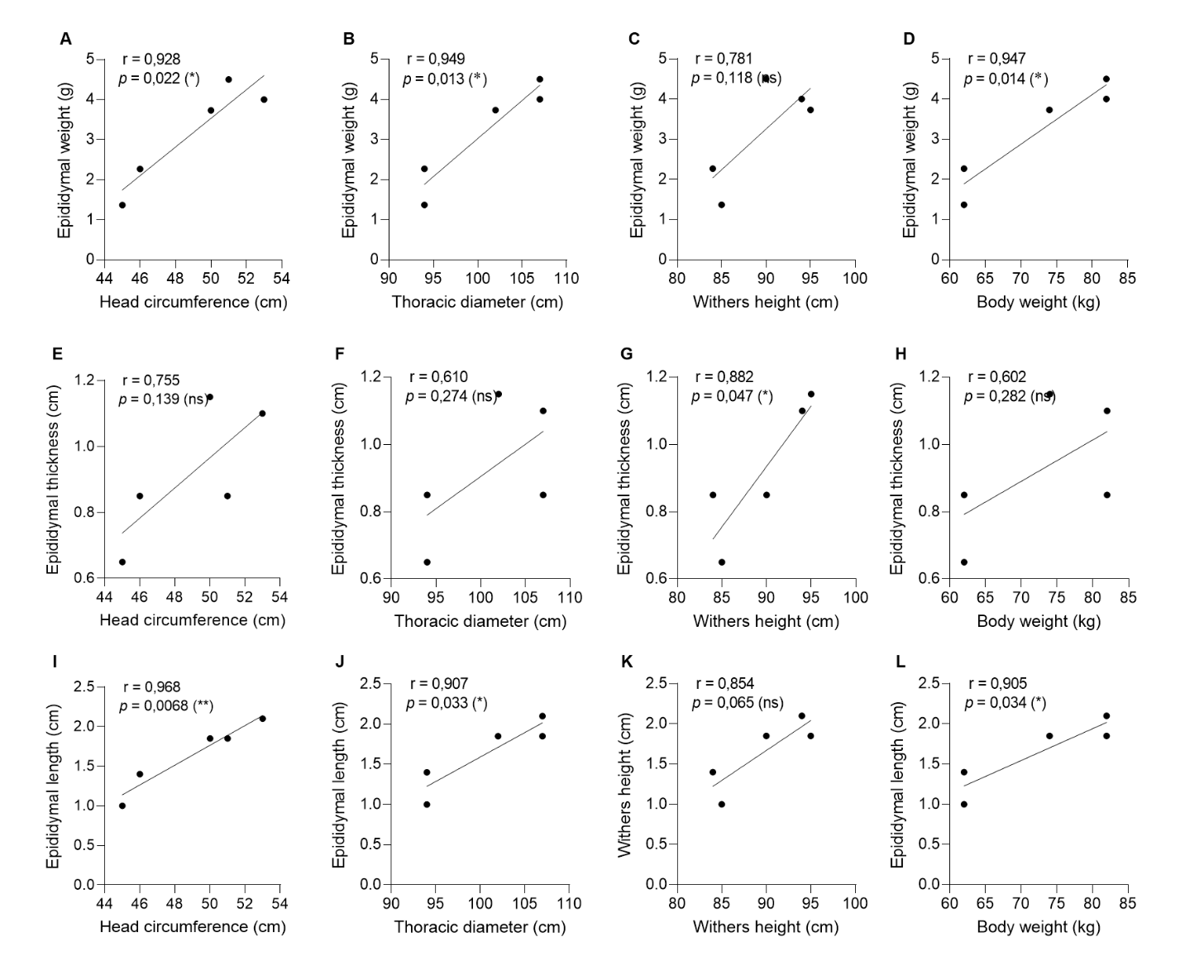


**Figure 2** - Correlations between body biometric measurements and epididymal biometrics in adult male sambar deer. A-L) Correlations between epididymal weight (A-D), thickness (E-H) and length (I-L) with head circumference (A, E and I), thoracic diameter (B, F and J), withers height (C, G and K) and body weight (D, H and L) in adult male sambar deer. Legend: *p<0.05; **p<0.01.


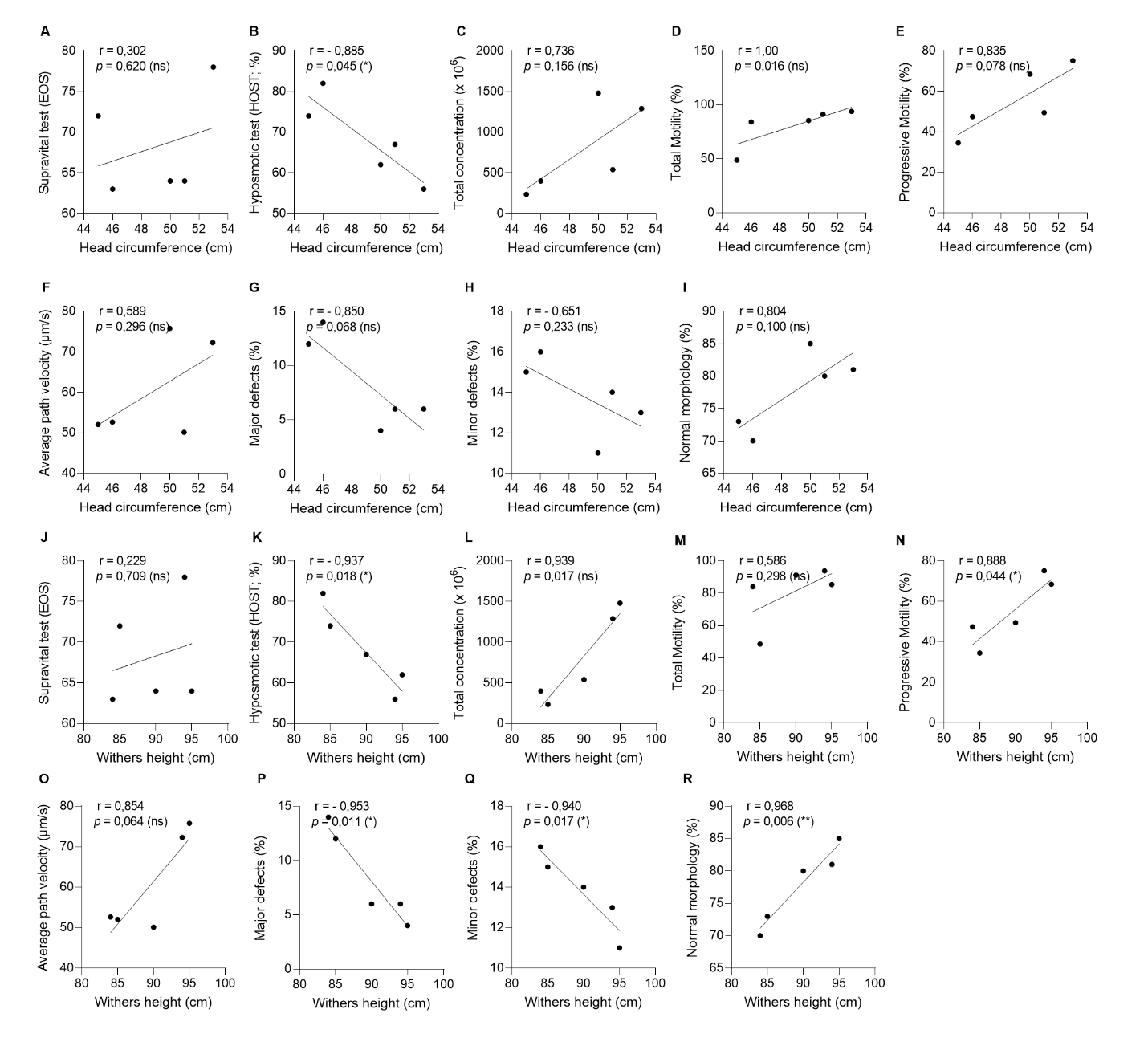


**Figure 3** – Correlations between body biometric measurements and sperm parameters in adult male sambar deer. (A-R) Correlations between head circumference (A-I) and withers height (J-R), supravital test (A and J), hyposmotic test (B and K), total concentration (C and L), total motility (D and M), progressive motility (E and N), average path velocity (F and O), major defects (G and P), minor defects (H and Q), and sperm with normal morphology (I and R) in adult male sambar deer. Legend: *p<0.05; **p<0.01.


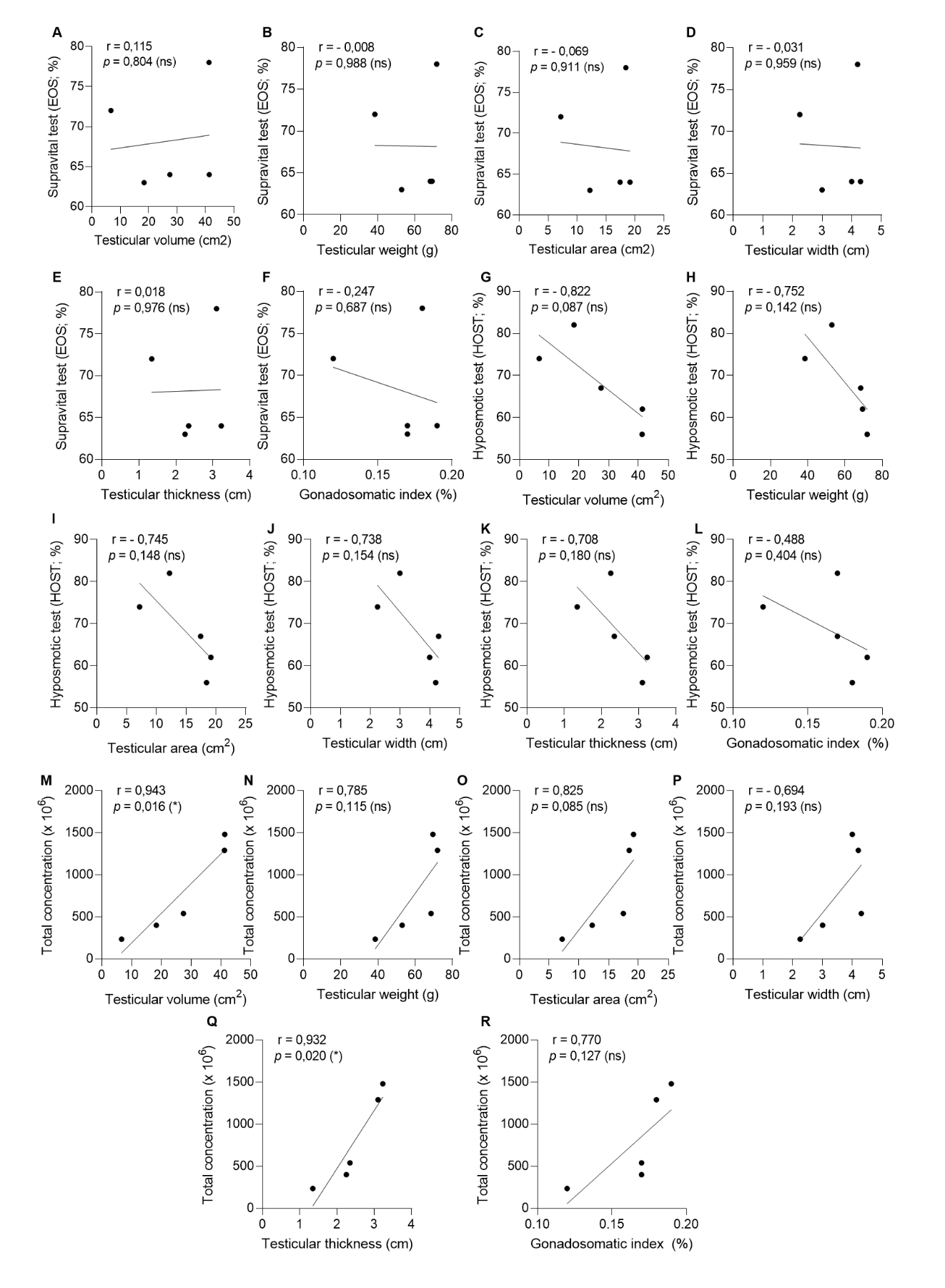


**Figure 4 –** Correlations between testicular biometric measurements and sperm parameters in adult male sambar deer. (A-R) Correlations between volume (A, G and M), weight (B, H and N), area (C, I and O), width (D, J and P), testicular thickness (E, K and Q), and gonadosomatic index (F, L and R) with the supravital test (A-F), hyposmotic test (G-L) and total concentration (M-R) in adult male sambar deer. Legend: *p<0.05.


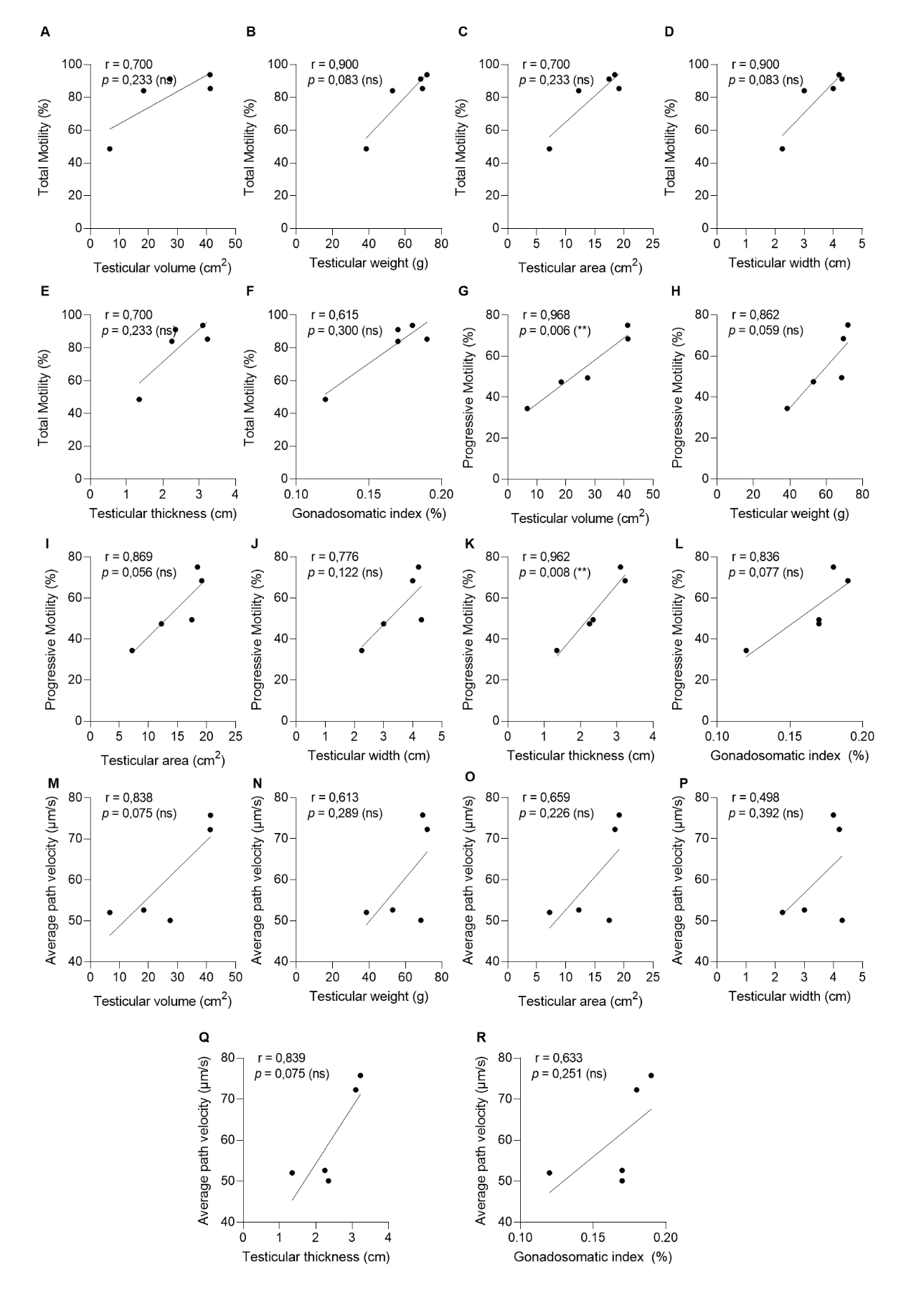


**Figure 5** – Correlations between testicular biometric measurements and sperm parameters in adult male sambar deer. (A-R) Correlations between volume (A, G and M), weight (B, H and N), area (C, I and O), width (D, J and P), testicular thickness (E, K and Q), and gonadosomatic index (F, L and R) with the total motility (A-F), progressive motility (G-L) and average path velocity (M-R) in adult male sambar deer. Legend: **p<0.01.


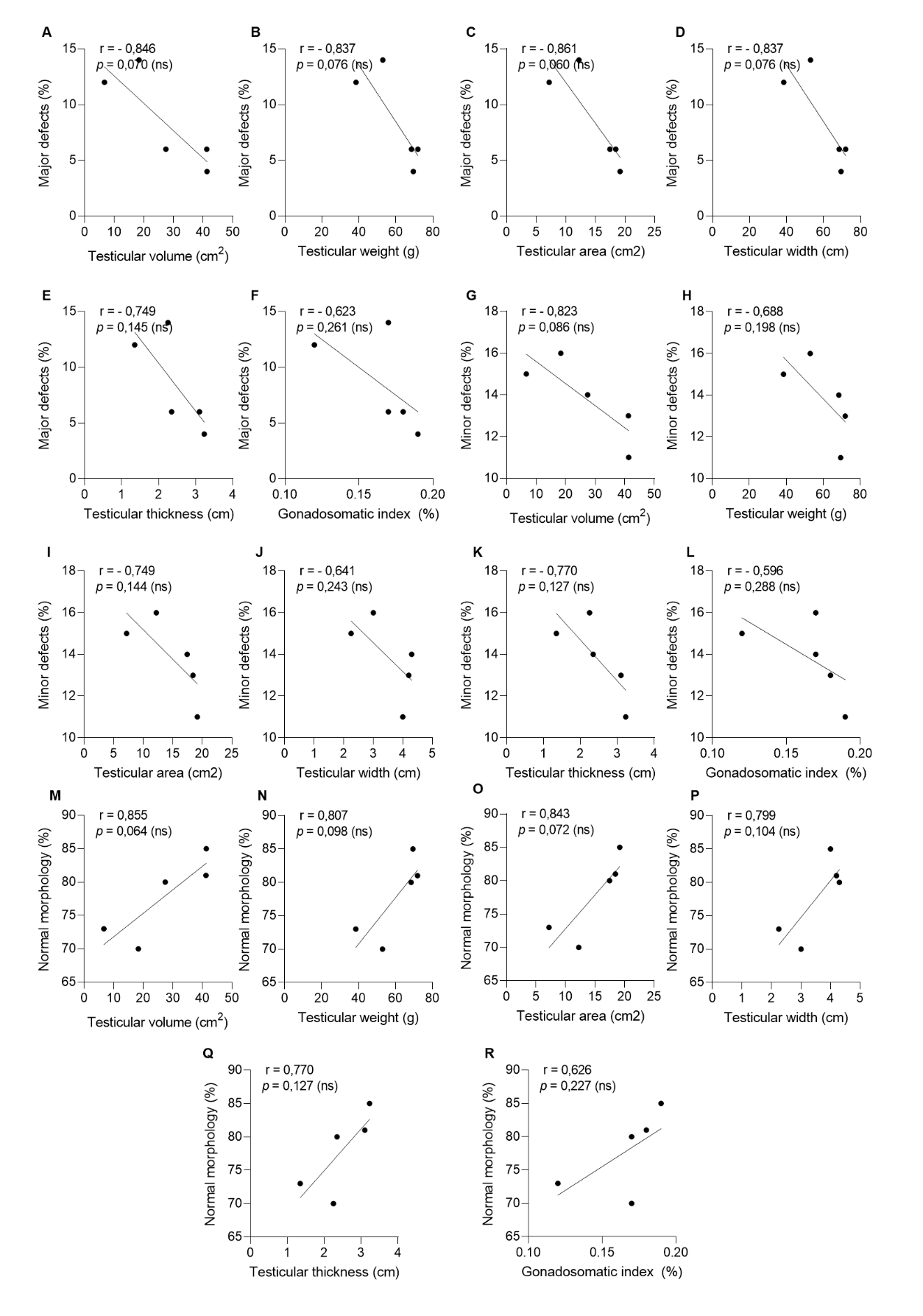


**Figure 6** - Correlations between testicular biometric measurements and sperm parameters in adult male sambar deer. (A-R) Correlations between volume (A, G and M), weight (B, H and N), area (C, I and O), width (D, J and P), testicular thickness (E, K and Q), and gonadosomatic index (F, L and R) with the major defects (A-F), minor defects (G-L) and normal morphology (M-R) in adult male sambar deer.


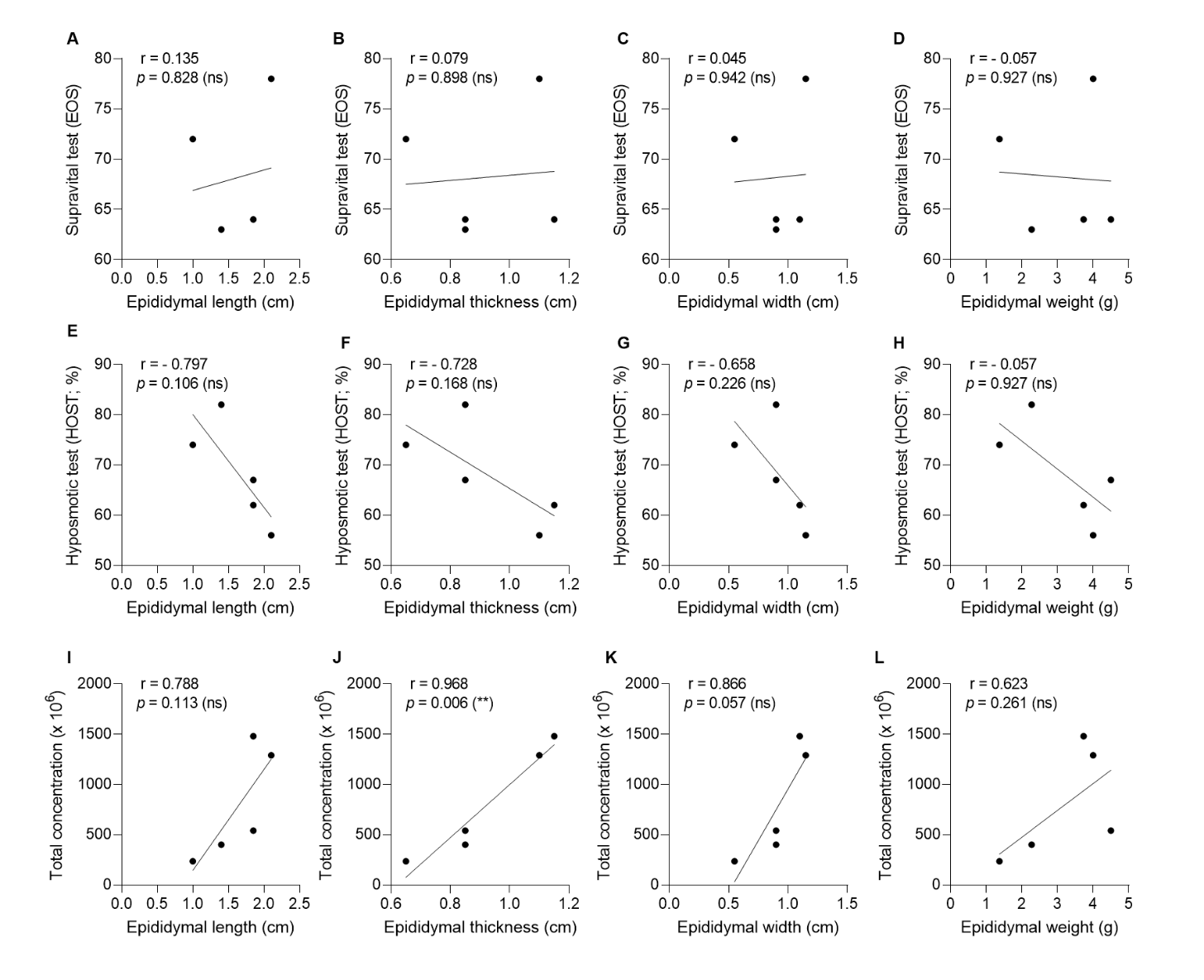


**Figure 7** – Correlations between epididymal biometric measurements and sperm parameters in adult male sambar deer. A-L) Correlations between epididymal length (A, E and I), thickness (B, F and J), width (C, G and K) and weight (D, H and L) with supravital test (A-D), hyposmotic test (E-H), and total concentration (I-L) in adult male sambar deer. Legend: **p<0.01.


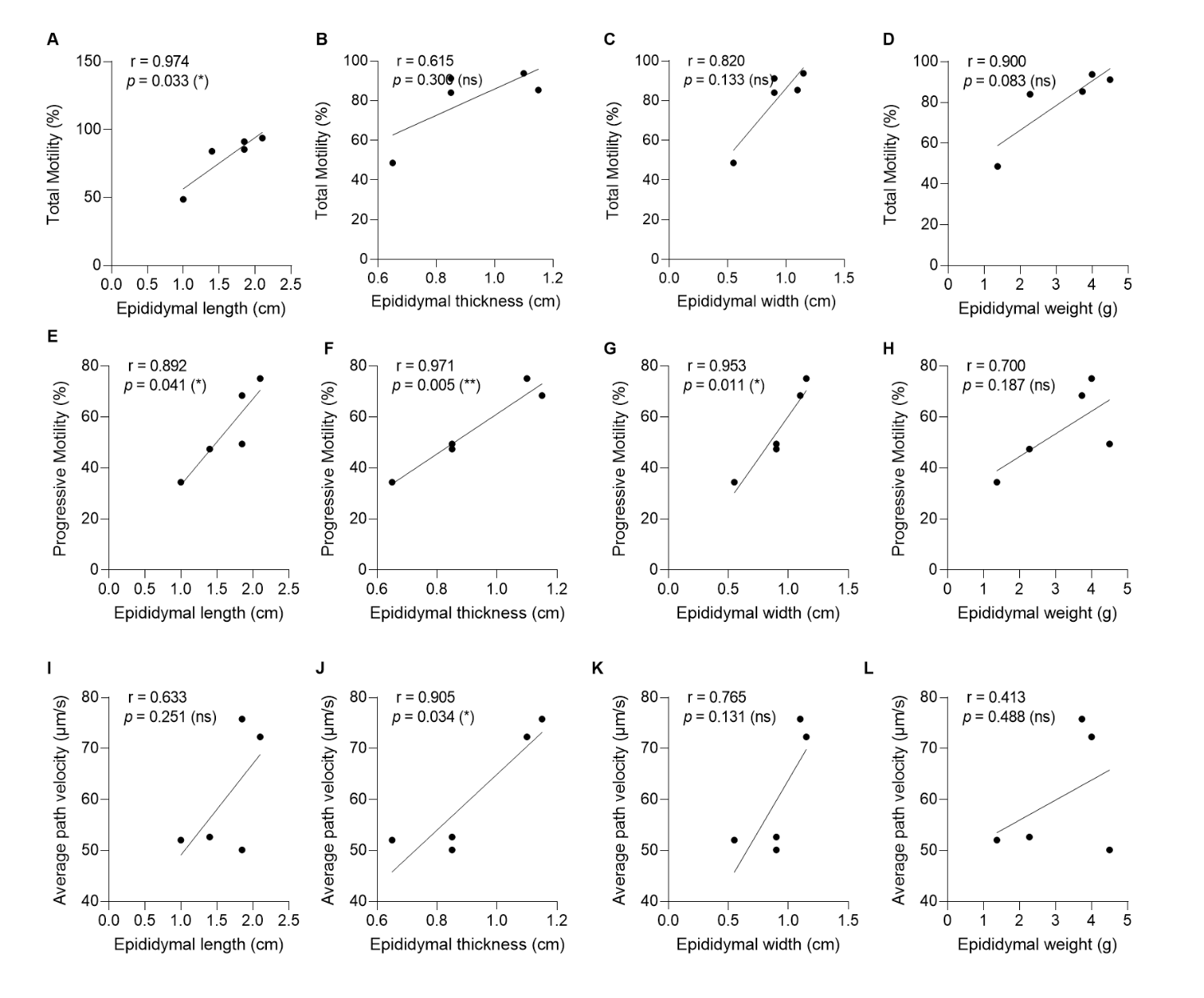


**Figure 8 –** Correlations between epididymal biometric measurements and sperm parameters in adult male sambar deer. A-L) Correlations between epididymal length (A, E and I), thickness (B, F and J), width (C, G and K) and weight (D, H and L) with total motility (A-D), progressive motility (E-H), and average path velocity (I-L) in adult male sambar deer. Legend: *p<0.05; **p<0.01.


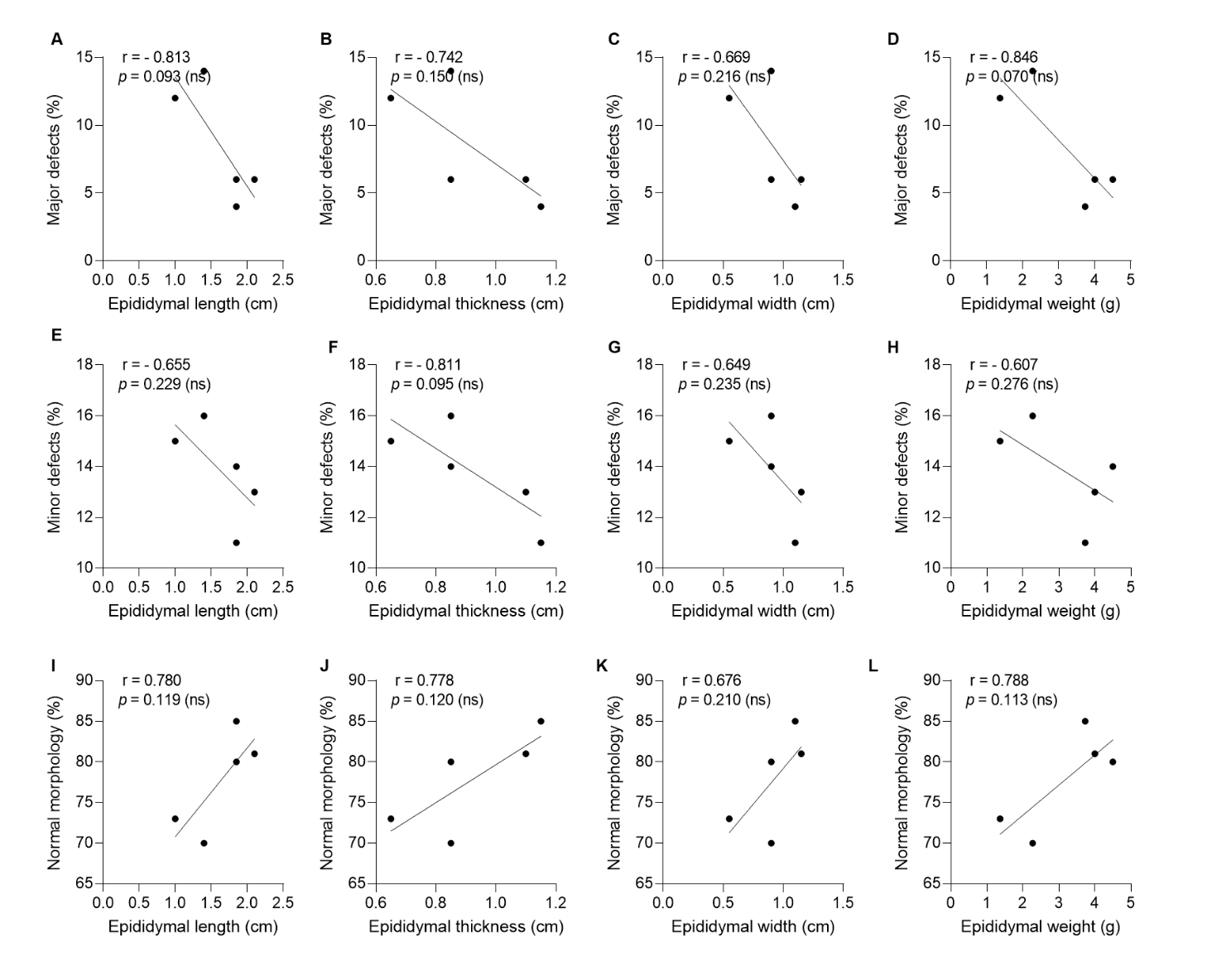


**Figure 9 -** Correlations between epididymal biometric measurements and sperm parameters in adult male sambar deer. A-L) Correlations between epididymal length (A, E and I), thickness (B, F and J), width (C, G and K) and weight (D, H and L) with major defects (A-D), minor defects (E-H), and normal morphology (I-L) in adult male sambar deer.


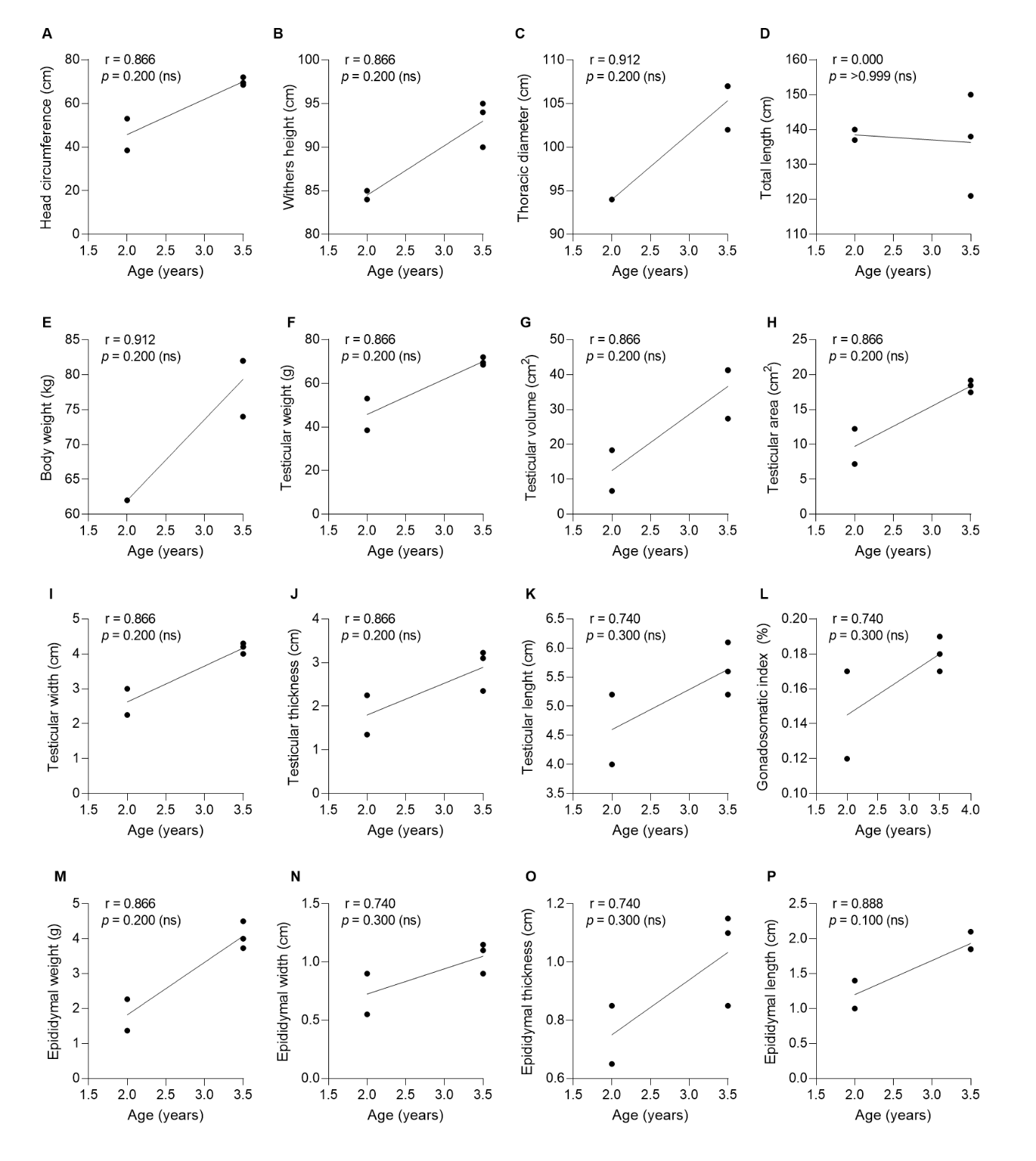


**Figure 10 -** Correlations between age and body biometrics, testicular and epididymal parameters in male sambar deer (n = 5). A-P) Correlations between age and head circumference (A), Withers height (B), Thoracic diameter (C), Total length (D), Body weight (E), Testicular weight (F), Testicular volume (G), Testicular area (H), Testicular width (I), Testicular thickness (J), Testicular length (K), Gonadosomatic index (L), Epididymal weight (M), Epididymal width (N), Epididymal thickness (O) and Epididymal length (P).


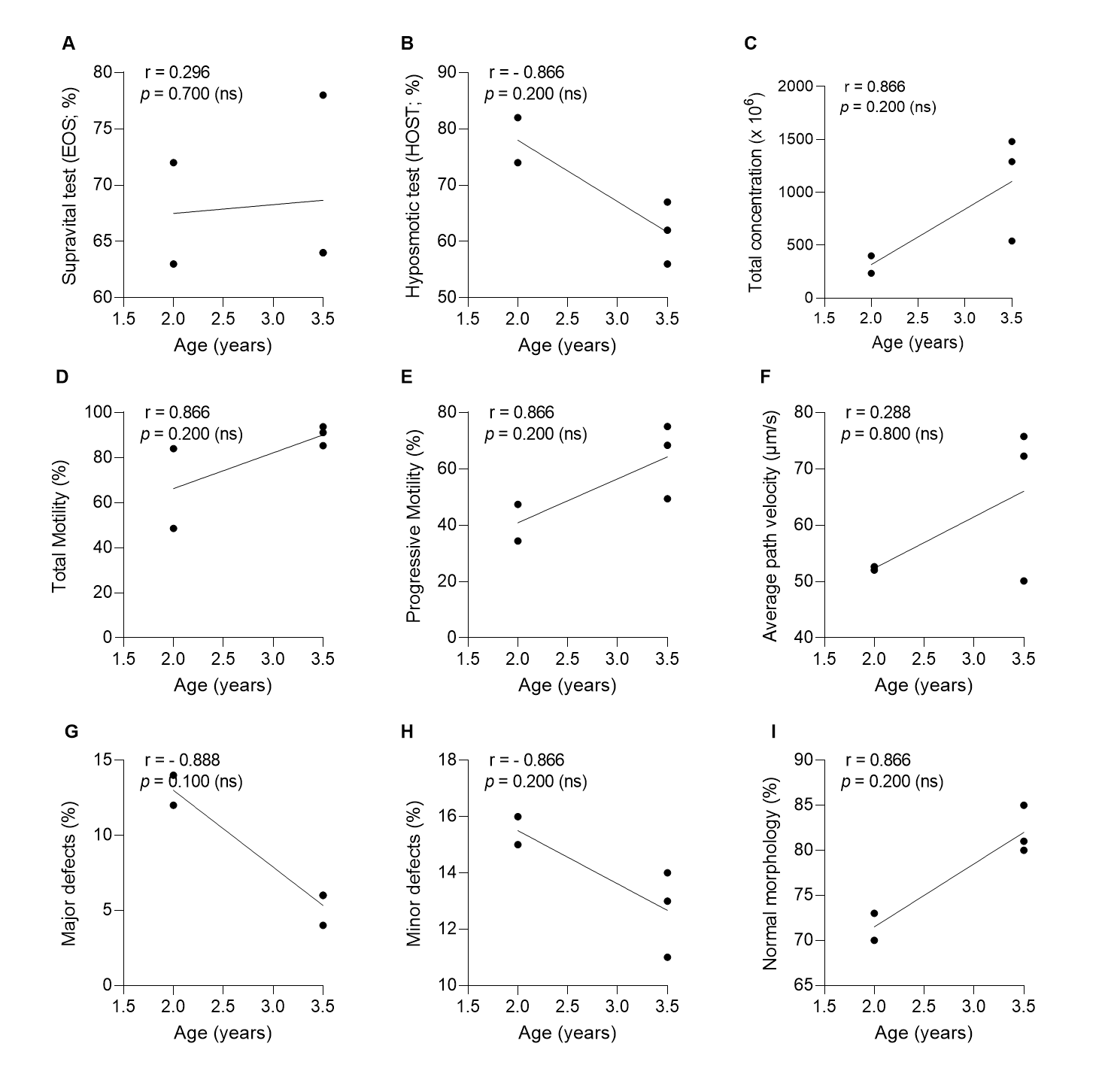


**Figure 11** **-** Correlations between age and sperm parameters in male sambar deer (n = 5). A-P) Correlations between age and head Supravital test (EOS) (A), Hyposmotic test (HOST) (B), Total concentration (C), Total motility (D), Progressive motility (E), Average path velocity (F), Major defects (G) Minor defects (H) and Normal morphology (I).
